# Supplementary material for: Bioactive mineralized small intestinal submucosa acellular matrix/PMMA bone cement for vertebral bone regeneration
Source: Regen Biomater. 2023 May 11;10:rbad040. doi: 10.1093/rb/rbad040 (PMC10224805; doi:10.1093/rb/rbad040)
Supplement: rbad040_Supplementary_Data [file rbad040_supplementary_data.zip › GA-Publication License Apr-19-2023.pdf]

49 Spadina Ave. Suite 200
Toronto ON M5V 2J1 Canada
www.biorender.com

Confirmation of Publication and Licensing Rights

April 19th, 2023
Science Suite Inc.

Subscription:	*Student Plan*
Agreement number:	*AJ259GBVP2*
Journal name:	*Guoqiang Jiang*

To whom this may concern,

This document is to confirm that Xinbao Miao has been granted a license to use the BioRender content, including icons, templates and other original artwork, appearing in the attached completed graphic pursuant to BioRender's Academic License Terms. This license permits BioRender content to be sublicensed for use in journal publications.

All rights and ownership of BioRender content are reserved by BioRender. All completed graphics must be accompanied by the following citation: "Created with BioRender.com".

BioRender content included in the completed graphic is not licensed for any commercial uses beyond publication in a journal. For any commercial use of this figure, users may, if allowed, recreate it in BioRender under an Industry BioRender Plan.

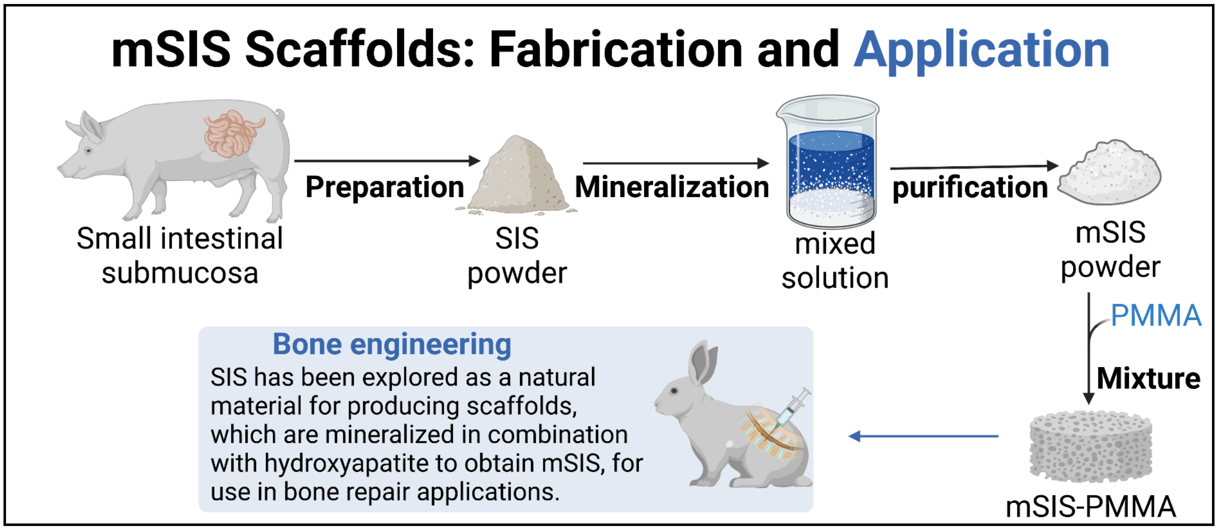

For any questions regarding this document, or other questions about publishing with BioRender refer to our BioRender Publication Guide, or contact BioRender Support at support@biorender.com.
