# Supplementary material for: Bioactive mineralized small intestinal submucosa acellular matrix/PMMA bone cement for vertebral bone regeneration
Source: Regen Biomater. 2023 May 11;10:rbad040. doi: 10.1093/rb/rbad040 (PMC10224805; doi:10.1093/rb/rbad040)
Supplement: rbad040_Supplementary_Data [file rbad040_supplementary_data.zip › Similarity Report.pdf]

PAPER NAME

0330173210667.docx

WORD COUNT

7393 Words

CHARACTER COUNT

42758 Characters

PAGE COUNT

30 Pages

FILE SIZE

5.9MB

SUBMISSION DATE

Mar 30, 2023 5:38 PM GMT+8

REPORT DATE

Mar 30, 2023 5:39 PM GMT+8

7% Overall Similarity

The combined total of all matches, including overlapping sources, for each database.

- 6% Internet database
- 6% Publications database
- Crossref database
- Crossref Posted Content database
- 0% Submitted Works database

Excluded from Similarity Report

- Bibliographic material
- Small Matches (Less then 8 words)

Bioactive mineralized small intestinal submucosa acellular matrix/PMMA bone cement for vertebral bone regeneration

Xinbao Miao [a#], Shuhui Yang [c#], Jinjin Zhu [b#], Zhe Gong [b], Dongze Wu [a], Juncong Hong [d], Kaiwen Cai [a], Jiying Wang [b], Xiangqian Fang [b*], Jiye Lu [a*], Guoqiang Jiang [a*]

[#] This work has been contributed equally by these authors.

[a] The Affiliated Hospital of Medical School, Ningbo University, Ningbo 315020, Zhejiang, China

[b] Department of Orthopedic Surgery, Sir Run Shaw Hospital, Zhejiang University School of Medicine & Key Laboratory of Musculoskeletal System, 3 East Qingchun Road, Hangzhou 310016, Zhejiang Province, China

Degeneration and Regeneration Translational Research of Zhejiang, Hangzhou 310016, China

[c] School of Materials Science and Engineering, Zhejiang-Mauritius Joint Research Center for Biomaterials and Tissue Engineering Zhejiang Sci-Tech University, Hangzhou, 310018, China

[d] Department of Anesthesiology, The First People's Hospital of Linping District, Hangzhou 311100, China

* Corresponding authors:

Guoqiang Jiang, E-mail: jiangguoqiang@nbu.edu.cn

Jiye Lu, E-mail: lucian0301@163.com

Xiangqian Fang, E-mail: orthofxq@zju.edu.cn

Abstract: Polymethylmethacrylate (PMMA) bone cement extensively utilized for the treatment of osteoporotic vertebral compression fractures due to its exceptional handleability and mechanical properties. Nevertheless, the clinical application of PMMA bone cement is restricted by its poor bioactivity and excessively high modulus of elasticity. Herein, mineralized small intestinal submucosa (mSIS) was incorporated into PMMA to prepare a partially degradable bone cement (mSIS–PMMA) that provided suitable compressive strength and reduced elastic modulus compared to pure PMMA. The ability of mSIS-PMMA bone

cement to promote the attachment, proliferation, and osteogenic differentiation of bone marrow mesenchymal stem cells was shown through cellular experiments carried out in vitro, and an animal osteoporosis model validated its potential to improve osseointegration. Considering these benefits, mSIS-PMMA bone cement shows promising potential as an injectable biomaterial for orthopedic procedures that require bone augmentation.

Keywords: PMMA bone cement, mineralized small intestinal submucosa, mechanical properties, osteogenic differentiation, osseointegration

1. Introduction

As the global population ages, osteoporosis is becoming increasingly prevalent [1]. Osteoporosis is a condition of the bones characterized by decreased bone density and compromised bone architecture, leading to a higher susceptibility to bone fractures [2,3]. The most prevalent form of fracture caused by osteoporosis is osteoporotic vertebral compression fracture (OVCF) [4,5], with ~1.4 million new cases estimated each year globally [6]. OVCF is primarily characterized by severe low back pain, which can severely limit mobility, significantly diminish the patient's quality of existence, and even pose a risk of fatality [7,8]. Percutaneous vertebroplasty and kyphoplasty are common minimally invasive surgical treatments for OVCF [9,10]. In clinical practice, polymethylmethacrylate (PMMA) is the most commonly utilized bone cement [11]; its good handleability and rapid dispersion after injection ensure rapid fracture stabilization and pain relief. Additionally, PMMA bone cement can provide immediate support after implantation, allowing patients to have early mobility [12]. However, PMMA bone cement has several disadvantages. The greater elasticity modulus of PMMA in comparison to the cancellous bone surrounding it can lead to stress shielding, which has the potential to cause secondary fractures in the vertebrae adjacent to the site where PMMA is applied [13]. Moreover, PMMA is biologically inert and nondegradable within the body and does not promote osteocyte growth. Consequently, the adhesion of the

bone cement to the adjacent bone interface is weak [14]. Therefore, the bone cement may be displaced at the surgical site [15]. Despite these limitations, PMMA bone cement is an excellent base material, and extensive research has been conducted to reduce its elastic modulus and impart bioactivity to meet the clinical needs of OVCF treatment.

Incorporating active materials into PMMA bone cement can help address these limitations [16]. Porcine small intestinal submucosa (SIS) is a natural substance composed of collagen and various bioactive compounds, such as growth factors that occur naturally in tissues, providing structural support to cells [17-19]. These properties, along with its good biocompatibility and 3D microenvironment [20], make SIS an attractive bioactive substance for bone regeneration. SIS-doped PMMA bone cement enhanced cellular growth and bone-forming ability *in vitro* and improved osseointegration in a vertebral defect animal model. It also had a lower elastic modulus than that of commercially available pure PMMA [21]. Research on SIS scaffolds has demonstrated their capacity to facilitate adherence, growth and differentiation of various cell lineages, such as bone marrow mesenchymal stem cells (BMSCs), osteoblasts, and fibroblasts, resulting in accelerated bone regeneration. These findings suggest that SIS scaffolds exhibit both osteoinductive and osteoconductive properties [22]. In addition, SIS can serve as a scaffold for the formation and growth of nano-hydroxyapatite (nHA) under physiological conditions, resulting in the production of mineralized SIS (mSIS) with the potential to promote neo-osteogenesis [23]. This suggests that mSIS can be used to create scaffolds that imitate the composition and structure of natural bone. Enhancing the osteogenic properties of scaffolds can be achieved through the biomineralization process that promotes the adherence of nHA to the surface of collagen scaffolds [24]. Compared with conventional PMMA bone cement, nHA-coated PMMA has superior biocompatibility and significantly enhances cell attachment [25].

We hypothesized that combining mSIS and PMMA could improve PMMA's physical and biological characteristics, yielding a bone cement with a reduced elastic modulus and

increased bioactivity compared to those of pure PMMA bone cement. This mSIS–PMMA composite would also maintain the favorable support properties and mechanical flexibility of PMMA. Accordingly, mSIS was prepared using *in vitro* biomimetic mineralization and used as an active filler to modify PMMA bone cement to produce partially biodegradable mSIS–PMMA bone cement. PMMA bone cement was tested for mechanical and biological properties before and after modification with mSIS. In addition, the mechanism by which mSIS affects osteogenesis was investigated by assessing the gene and protein expression quantities. Finally, the effect of adding mSIS powder to PMMA bone cement on *in vivo* bone integration was studied in osteoporotic rabbits.

2. Materials and methods

2.1 Materials synthesis

Fresh porcine jejunum was mechanically dissociated, defatted, decontaminated, enzymatically digested, and lyophilized to obtain dried SIS. Thereafter, the dried SIS was pulverized using a cryo-mill, followed by sifting the resulting powder through a 200-mesh test sieve to obtain powdered SIS. Next, an acidic SIS suspension was prepared by adding deionized water to the SIS powder, followed by the addition of phosphate (7558-80-7; Aladdin, Shanghai, China) and soluble calcium (10043-52-4; Aladdin) solutions at a Ca:P ion ratio of 5:3. Afterward, a sodium hydroxide solution (1310-73-2; Aladdin) was added slowly to neutralize the solution and obtain the mSIS precipitate. After centrifugation and sterile water washing, the mSIS precipitate was collected and lyophilized. The resulting mSIS powder was obtained by grinding through the aforementioned screen. The range of particle sizes in the mSIS powder was then determined by analyzing the particle size distribution using a laser-based method.

mSIS was combined with PMMA powder, and liquid MMA was added at a solid to liquid ratio of 2.00:2.27 g/mL. The mSIS–PMMA bone cement was prepared after being fully

mixed. Table 1 shows the Mendec Spine® PMMA bone cement composition before and after modification.

Table 1. Compositions of mSIS–PMMA bone cement with different mSIS:PMMA ratios (values in g).

Component	Constituent	0–100	5–95	10–90	15–85	5–100	10–100	15–100	20–100
Powder	mSIS	0.00	1.00	2.00	3.00	0.95	1.83	2.61	3.33
	Polymethylmethacrylate	13.50	12.83	12.16	11.49	12.86	12.27	11.74	11.25
	Barium sulphate	6.00	5.70	5.40	5.10	5.71	5.45	5.22	5.00
	Benzoyl peroxide	0.50	0.47	0.44	0.41	0.48	0.45	0.43	0.42

2.2 Physical characterization tests

The four stages of the cement curing process (mixing, waiting, working, and setting) were evaluated at each mixing weight ratio. This procedure was performed in triplicate to obtain an average value.

Fourier-transform infrared (FTIR) analysis was performed on KBr disks of cured PMMA and mSIS–PMMA (mass ratio of mSIS:PMMA = 15:85) bone cement. To confirm the attachment of nHA to the SIS, mSIS (10 mg) morphology was examined by transmission electron microscopy (TEM). Specimen cross-sections were imaged using a scanning electron microscope (SEM). Rectangular areas of 400 μm^2 were randomly selected and analyzed for the Ca:P ion ratio, employing energy dispersive X-ray spectroscopy (EDS).

PMMA and mSIS–PMMA bone cement samples, with varying mSIS:PMMA ratios and dimensions of 6 mm × 6 mm × 12 mm, were tested for strength under compression and

their ability to withstand deformation using a mechanical tester with a displacement velocity of 20 mm/min. Three specimens of each mixing weight ratio were tested to obtain an average value to ensure measurement accuracy. In addition, three distinct areas of each sample were chosen at random and measured using a water contact angle measurement system to determine the average static contact angle. Combined with previous tests, the proportion suitable for subsequent tests is screened.

The PMMA and mSIS–PMMA specimens (with a mass ratio of mSIS:PMMA = 15:85) were weighed and exposed to a degradation examination by submerging them in simulated body fluid (SBF). The samples were extracted from the SBF after 14 and 28 days for subsequent drying and weighing to measure degradation. The degradation rate (DR) was calculated as follows:

$$DR\ (\%) = \left(\frac{m1-m2}{m1}\right) \times 100,$$

where $m1$ and $m2$ are the masses of the bone cement samples before and after the degradation test, respectively.

2.3 Biological characterization tests

To conduct cell culture and proliferation tests, the surfaces of the different bone cements (10 mm diameter, 1 mm thickness, n = 5) were seeded with mouse BMSCs (MUBMX-01001; OriCell, Guangzhou, China) with a consistent initial cell seeding density of 6.5×10^3 cells/cm^2 and allowed to incubate in Dulbecco's Modified Eagle Medium (MUXMX-90011; OriCell) for 7 days. Cell proliferation was assessed by adding a CCK-8 working solution (HY-K0301; MedChemExpress, Princeton, NJ, USA) to the culture medium on days 1, 3, 5, and 7. Optical density measurement at 450 nm was performed using a microplate reader on the supernatant after a 2-hour dark incubation of the samples.

Following 7 days of incubation in a proliferation medium, cell viability on the BMSC-loaded bone cement disks was evaluated using calcein/PI (C2015S; Beyotime, Beijing, China). Stained cells were imaged and counted under a laser microscope. To examine BMSC morphology, the specimens underwent sequential dehydration using increasing ethanol strengths from 30 to 100%. The specimens were soaked in tert-butanol (T119717; Aladdin) overnight at 4 °C and freeze-dried for SEM observation.

To perform immunofluorescence staining, after proliferation culturing for 1, 3, 5, and 7 days and osteogenic induction culturing for 21 days (MUXMX-90021; OriCell), the BMSC-loaded disks were incubated with 100 nM rhodamine-phalloidin (CA1610; Solarbio, Beijing, China) following fixation, permeabilization, and blocking. After the above procedures, the specimens were treated with 4′ , 6-diamidino-2-phenylindole (DAPI; C0065, Solarbio) for staining, and subsequently analyzed using laser confocal microscop.

BMSCs were subjected to 21 days of bone-forming differentiation by culturing them in a medium that promotes this type of differentiation. Quantitative assays for alkaline phosphatase (ALP) were performed by lysing differentiated cells with lysis buffer (P0013J; Beyotime). After adding the working and reaction termination solutions to the lysed differentiated cells, a microplate reader was used to detect the ALP enzymatic reaction signal at a wavelength of 405 nm. The differentiated BMSCs were immobilized using 4% paraformaldehyde and subsequently incubated with 1 mL of alizarin red staining solution (C0148S; Beyotime) for 5 min. Thereafter, the stained Ca on the bone cement was imaged using a scanner.

Reverse transcription polymerase chain reaction (RT-qPCR) was used to investigate the gene expression of differentiated BMSCs. Briefly, the cells were subjected to RNA extraction using a commercial kit (CW0584; CoWin Biosciences, Beijing, China). A FastQuant RT kit (AG11707; Accurate Biology, Changsha, China) was utilized to reverse transcribe RNA to cDNA. Subsequently, RT-qPCR was conducted using 5 ng of cDNA per

reaction, with the forward and reverse primers specified in Supplementary Table S1, following the SYBR Green method.

Protein blot analysis was performed on differentiated BMSCs by lysing samples in a radioimmunoprecipitation assay buffer. Protein specimens were subjected to electrophoresis on Bis-Tris Gel (GF1810-10; GeneFirst, Oxfordshire, UK) followed by their transfer onto PVDF membranes. Initially, the membranes were subjected to overnight incubation utilizing primary antibodies directed against osteocalcin (OCN; 1:1,000; AB10911; Sigma-Aldrich, St. Louis, MO, USA), ALP (1:1,000; Huaan, China), and β-actin (1:5,000; , Huaan, China). Afterward, the membranes were exposed to the appropriate secondary antibodies, and protein visualization was achieved by enhanced chemiluminescence.

In the rabbit osteoporosis model, adult female New Zealand white rabbits aged 5 months (Cixi Yanling Otter Farm, China; weight 3.02 + 0.25 kg, n = 10) were utilized following the approved protocol of the Animal Experimentation Committee of Ningbo University (11473). Dual-energy X-rays were used to evaluate the lumbar vertebrae bone mineral density of the rabbits 1 week before the surgery. After anesthesia, bilateral ovariectomies were performed on the rabbits. Intramuscular injections of dexamethasone sodium phosphate were administered at a dosage of 1.8 mg/day for 8 consecutive weeks from the second week after osteoporosis surgery, and bone density was periodically determined during this period.

Osteoporotic female rabbits were divided into two groups for percutaneous vertebroplasty surgery: one group received pure PMMA bone cement, while the other group received mSIS–PMMA bone cement. The L3 spinous process was penetrated using a 14G bone biopsy needle to a depth of 2–3 mm, after which 0.2 mL of bone cement was injected.

Six weeks after percutaneous vertebroplasty surgery, the vertebral bodies injected with bone cement were excised. Subsequently, micro-CT scans were performed on all specimens. For data analysis, 3D CT reconstructions were performed for each specimen under the same

conditions. A region of interest measuring 1.5 mm × 1.5 mm, located at roughly the same position in each specimen, was chosen for assessing the 3D microarchitecture of the trabecular bone, encompassing the trabecular number (Tb.N) and volumetric ratio (BV/TV) of trabeculae. The effectiveness of the cement's regenerative capacity was evaluated via histological scoring (Supplementary Table S2) at the bone-implant interfaces. This allowed for the assessment of tissue response and bone ingrowth resulting from the cement [26].

To prepare for histological staining and imaging, the vertebral specimens of the rabbits were dehydrated, encased in MMA, and sliced into 10-µm-thick sections. Four slices containing bone cement were randomly selected from each specimen and subjected to methylene blue and magenta and Masson staining, followed by observation using a slice scanner.

2.4 Statistical analyses

Values are expressed as the mean ± standard deviation (SD) of a minimum of three procedural replicates. Statistical analysis was was executed utilizing SPSS package 28.0.1.1 (SPSS Inc., Chicago, IL, USA). Normally distributed variables were evaluated using an independent-sample *t*-test or one-way analysis of variance (ANOVA). Non-normally distributed variables were evaluated using non-parametric methods combined with appropriate post hoc tests for the least significant difference. Statistical significance was inferred for differences at a threshold of $P < 0.05$.

3. Results

3.1. mSIS–PMMA cement composition and properties.

The mSIS powder had an average diameter of 163.1 µm, with particle size frequency analysis indicating that 10, 50 (median diameter), and 90% of the particles had diameters of 14.76, 97.44, and 404.9 µm, respectively (Figure 1A). The nanomorphology of the mSIS observed by TEM is shown in Figure 1B. The mSIS fibers had an approximate diameter of 100 nm, and their surface comprised a randomly dispersed crystalline material. In terms of

operability, mSIS–PMMA bone cement showed similar curing behavior to PMMA; its curing time gradually decreased with increasing mSIS fraction up to an mSIS/PMMA powder ratio of 15:85 (Figure 1C). mSIS–PMMA bone cement (mSIS:PMMA mass ratio = 15:85) FTIR spectra (Figure 1D) confirmed organic (SIS) and inorganic (nHA) phases via characteristic peaks: HPO_4^{2-} (876 cm^{-1}), HPO_4^{3-} (1,100 cm^{-1}), amide II (C-N stretching, 1640 cm^{-1}), and amide A (N-H stretching, 3,445 cm^{-1}). The EDS elemental analysis results presented in Figure 1E demonstrate a uniform dispersion of calcium (Ca) and phosphorus (P) throughout the mSIS-PMMA bone cement, with a ion ratio of Ca to P of 1.52. SEM images of the cross-sectional morphology (Figure 1F) showed that PMMA had a dense and smooth cross-section, while the mSIS–PMMA contained randomly distributed fibrous mSIS.

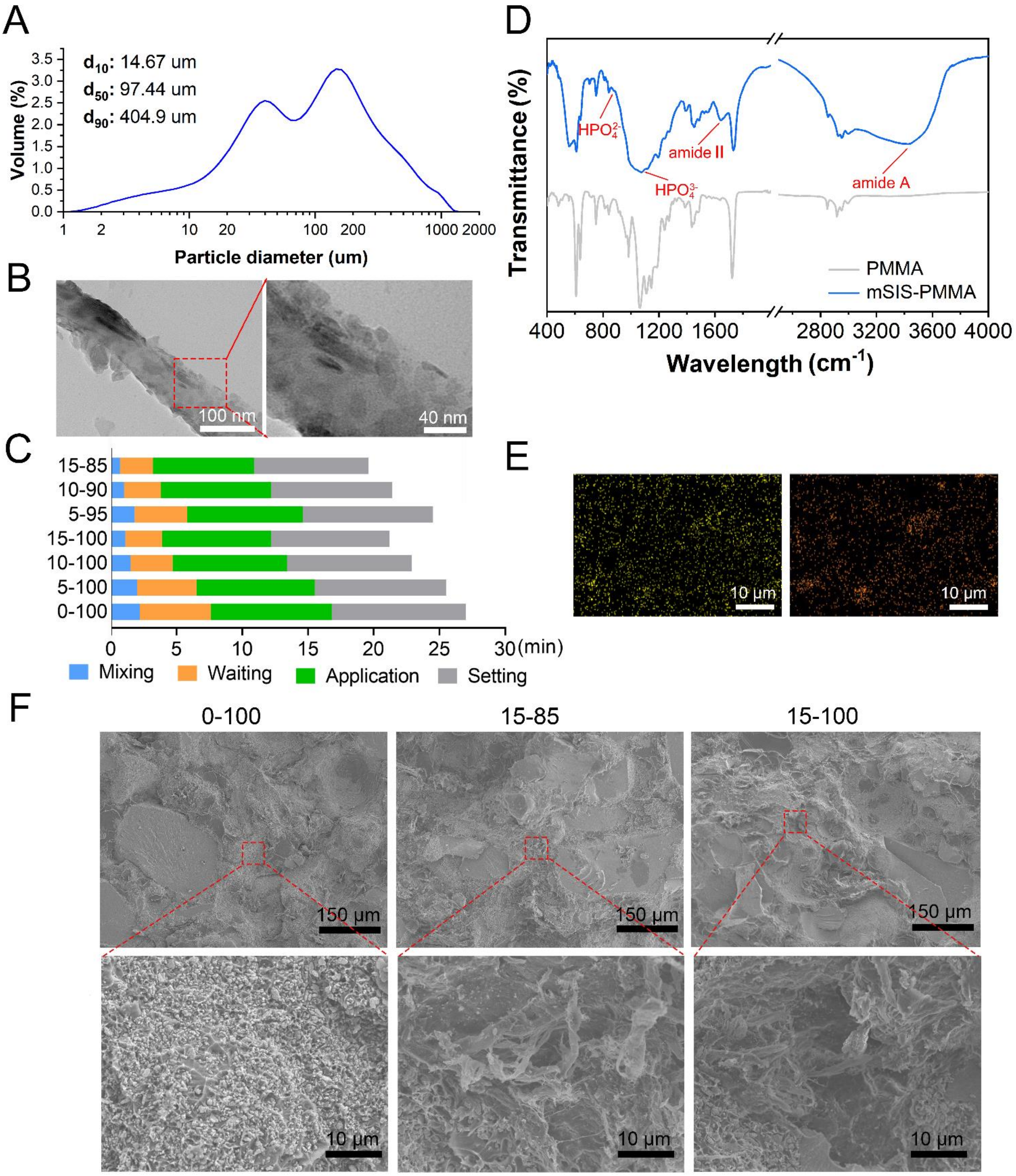

242 **Figure 1.** (A) Size range of mSIS particles. (B) TEM images showing the appearance of mSIS particles. (C) Processing times of bone cements with varying composition. (D) FTIR spectra. (E) Ca and P are distributed in mSIS-PMMA cement in yellow and orange, respectively. (F) SEM micro-morphologies of different mSIS-PMMA ratios.

Before reaching the yield point, the stress-strain curves of all materials exhibited comparable linear elastic behavior, as shown in Figure 2A during mechanical testing. The PMMA sample had a compressive strength of 109.66 ± 5.43 MPa, which decreased with increasing mSIS fraction. The compressive strengths of the bone cements with mSIS:PMMA ratios of 5:100, 10:100, 15:100, 5:95, 10:90, and 15:85 were 99.06 ± 3.2, 95.41 ± 2.7, 85.17 ± 2.9, 97.74 ± 4.9, 89.33 ± 3.3, and 79.21 ± 4.3 MPa, respectively (Figure 2B). The elastic modulus of PMMA was 2,281 ± 53 MPa, while the mSIS–PMMA bone cements with ratios of 15:85 and 15:100 had elastic moduli of 1,012 ± 93 and 1,195.8 ± 31 MPa, respectively, closer to the range for human cancellous bone (50–800 MPa). The elastic moduli of bone cements with mSIS:PMMA ratios of 5:100, 10:100, 5:95, and 10:90 were 1,858.84 ± 47, 1,484.52 ± 78, 1,763.19 ± 52, and 1,324.96 ± 77 MPa, respectively (Figure 2C).

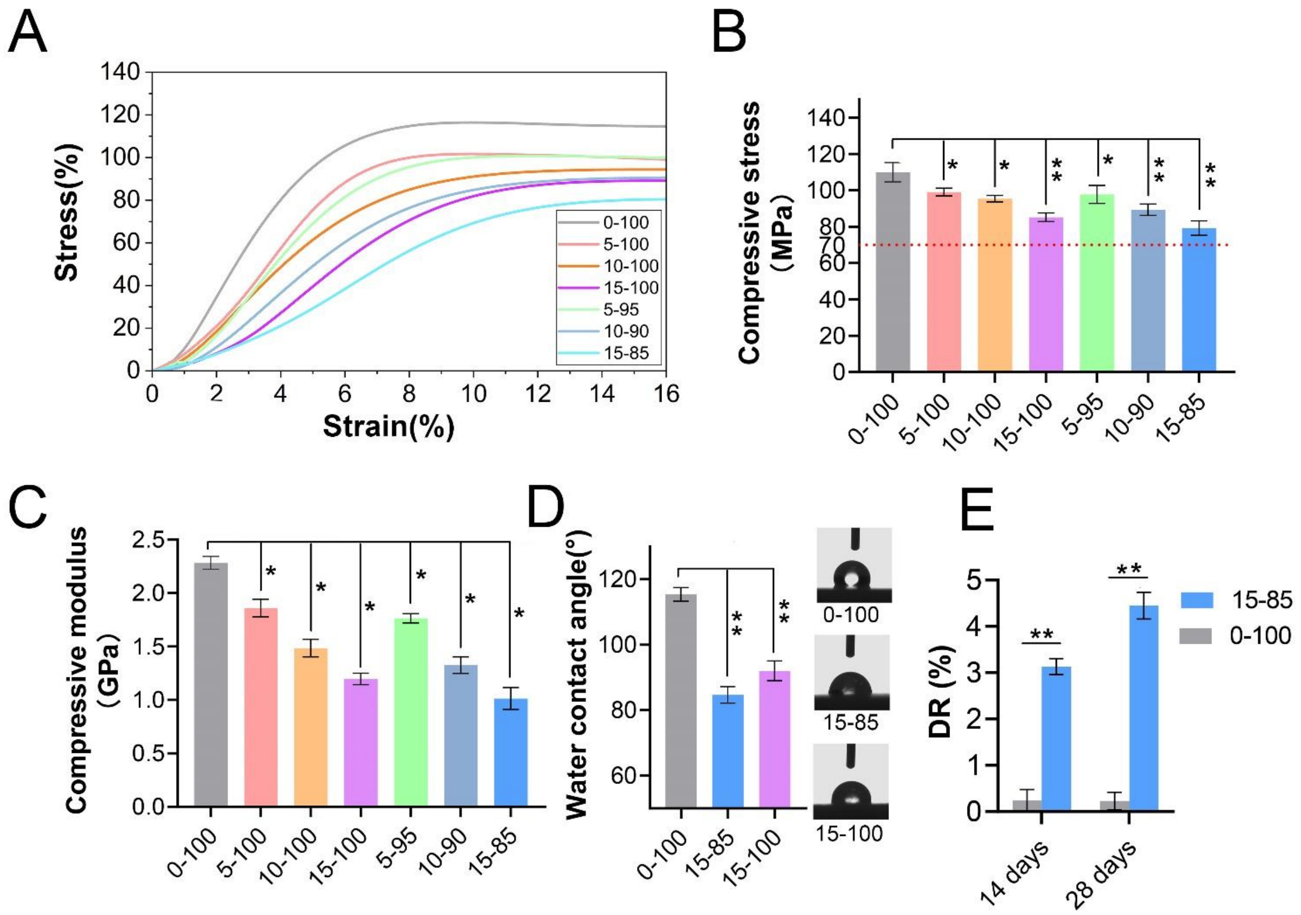

Figure 2. Properties related to the mechanics of mSIS–PMMA bone cement. (A) Stress–strain curves, (B) compressive strength, (C) compressive modulus, (D) water contact angle, and (E)

degradation degree (DR). Significance: *$P < 0.05$ and **$P < 0.01$ vs. 0–100 group. Error bars represent SD.

The water contact angle was 113 ± 3° for PMMA, typical for a hydrophobic polymeric material. The water contact angle of mSIS–PMMA bone cement decreased with increasing mSIS content to 85 ± 2° and 92 ± 3° for the 15:85 and 15:100 ratios, respectively (Figure 2D). Due to its hydrophilicity and mechanical characteristics, 15:85 mSIS–PMMA bone cement was chosen for further biological analyses. Figure 2E depicts the changes observed in PMMA and mSIS–PMMA bone cements (mass ratio of mSIS:PMMA = 15:85) after they were left to soak in SBF for 2 and 4 weeks. There was almost no observed mass change of PMMA in SBF. The mean DR of mSIS–PMMA on days 14 and 28 were 3.13 and 4.45%, respectively.

3.2. Impact of mSIS–PMMA bone cement on BMSCs

The calcein AM/PI staining of BMSCs cultured for 7 days on PMMA or mSIS–PMMA bone cements (Figure 3A) revealed that mSIS–PMMA had a higher number of viable cells (green) compared with that of PMMA. However, the number of dead cells (red) did not significantly differ, suggesting that mSIS–PMMA enhanced cell attachment and promoted cell viability. After 3 days of culture, CCK-8 tests showed significantly higher optical density values for cells on mSIS–PMMA compared with those on PMMA (Figure 3B). This indicates that mSIS–PMMA promoted cell proliferation to a greater extent than PMMA. Immunofluorescence staining revealed that after 1 day of culture, BMSCs showed similar attachment on all samples, but mSIS–PMMA supported cells with more and longer cellular pseudopods than those on PMMA. The cell densities on the surface of mSIS–PMMA were considerably higher than those on PMMA at days 5 and 7 of culture, as shown in Figure 3C. SEM imaging of the BMSC morphology on day 7 also showed that compared with PMMA, mSIS–PMMA had more cells on its surface; additionally, the cells were better-attached, with

expanded cell morphology and extended pseudopods (Figure 3D). This confirmed that mSIS–PMMA promoted BMSC attachment and proliferation better than PMMA. Quantitative and statistical analyses were performed on randomly selected cells on the bone cements using ImageJ software (NIH, Bethesda, MD, USA). BMSCs covered 1.3 times larger surface area on mSIS-PMMA than PMMA (Figure 3E).

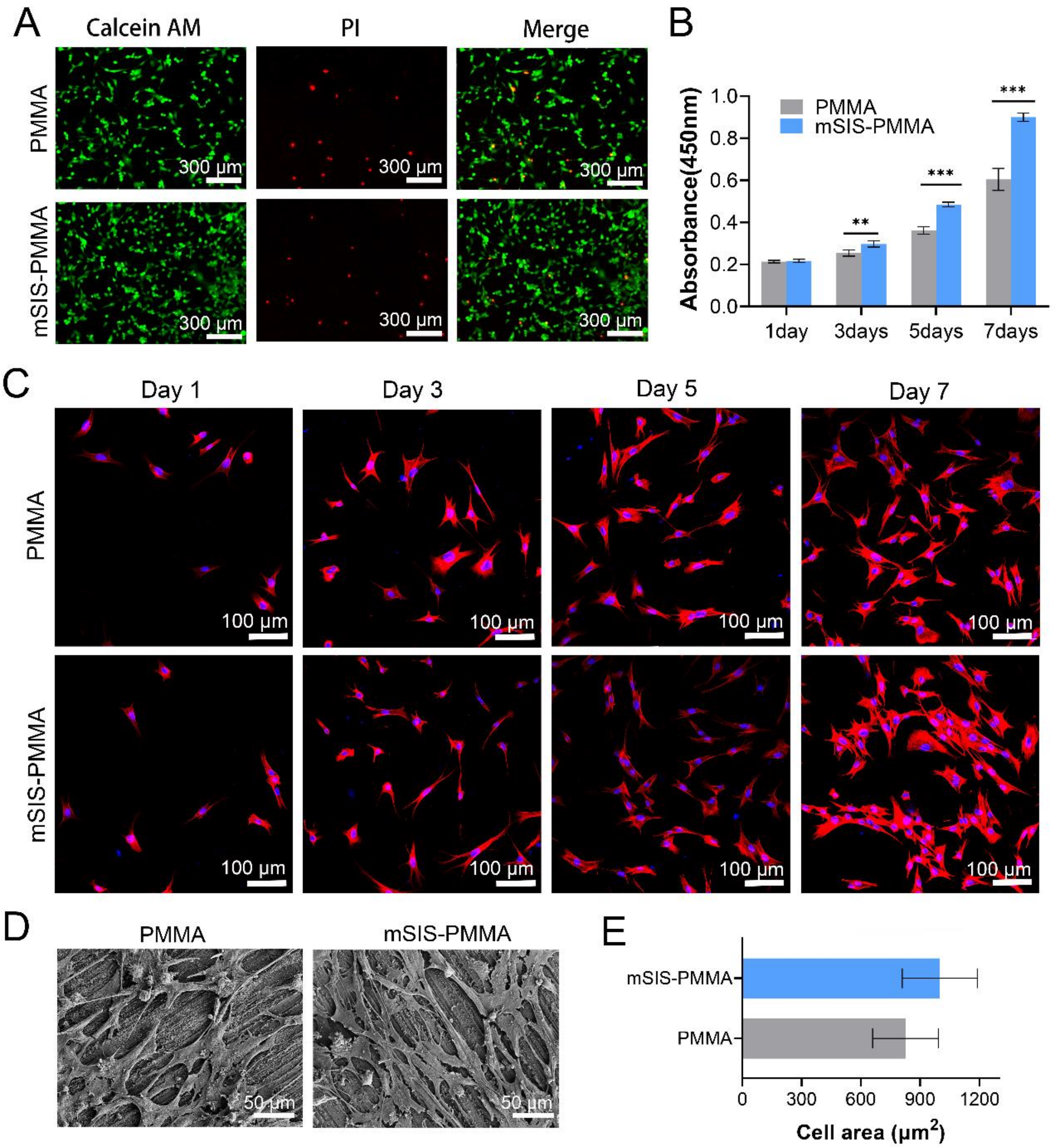

Figure 3. PMMA bone cement modification impact on cell bioactivity: (A) live/dead staining for BMSC viability; (B) BMSC optical density values of bone cement extracts; *n* = 5; **P* <

0.05, $**P < 0.01$, $***P < 0.001$. (C) BMSC morphology on days 1, 3, 5, and 7, (D) cell morphology observed by SEM, and (E) cell area on the bone cements.

3.3. Osteogenic differentiation of BMSCs on the bone cements

Osteogenically differentiated BMSCs were stained to reveal their cytoskeletal structure. Compared with the control, staining revealed higher cell density on mSIS–PMMA bone cement, more pronounced local cluster growth, more obvious fusion, and more extensive pseudopod extension and spreading (Figure 4A–C). ALP activity was utilized as an indicator to evaluate the osteogenic differentiation potential of mSIS-PMMA bone cement. mSIS-PMMA manifested a significant upsurge in ALP activity as compared to the control (Figure 4D). Thus, mSIS enhances BMSC osteogenic differentiation and stimulates increased ALP expression. In addition, the cells were stained with alizarin red to assess matrix mineralization (Ca nodules formed at the later stages of osteoblastic differentiation). The differentiation of BMSCs into osteoblasts on mSIS-PMMA bone cement was facilitated, as indicated by the occurrence of more irregular areas that were stained dark red in comparison to the control (Figure 4E).

The impact of mSIS on osteogenic gene and protein expression was also investigated. RT-qPCR showed that BMSCs on mSIS–PMMA had significantly higher levels of ALP, OCN, BMP2, Runx2, and Col-1 genes than those on PMMA. ALP and Runx2 levels were nearly tripled, while OCN levels were upregulated almost 7-fold (Figure 4F). Protein blot analysis revealed a significant increase in ALP and OCN protein expression in differentiated BMSCs cultured on mSIS–PMMA compared with the control (Figure 4G), suggesting that mSIS promoted osteogenic differentiation of BMSCs at both gene and protein levels.

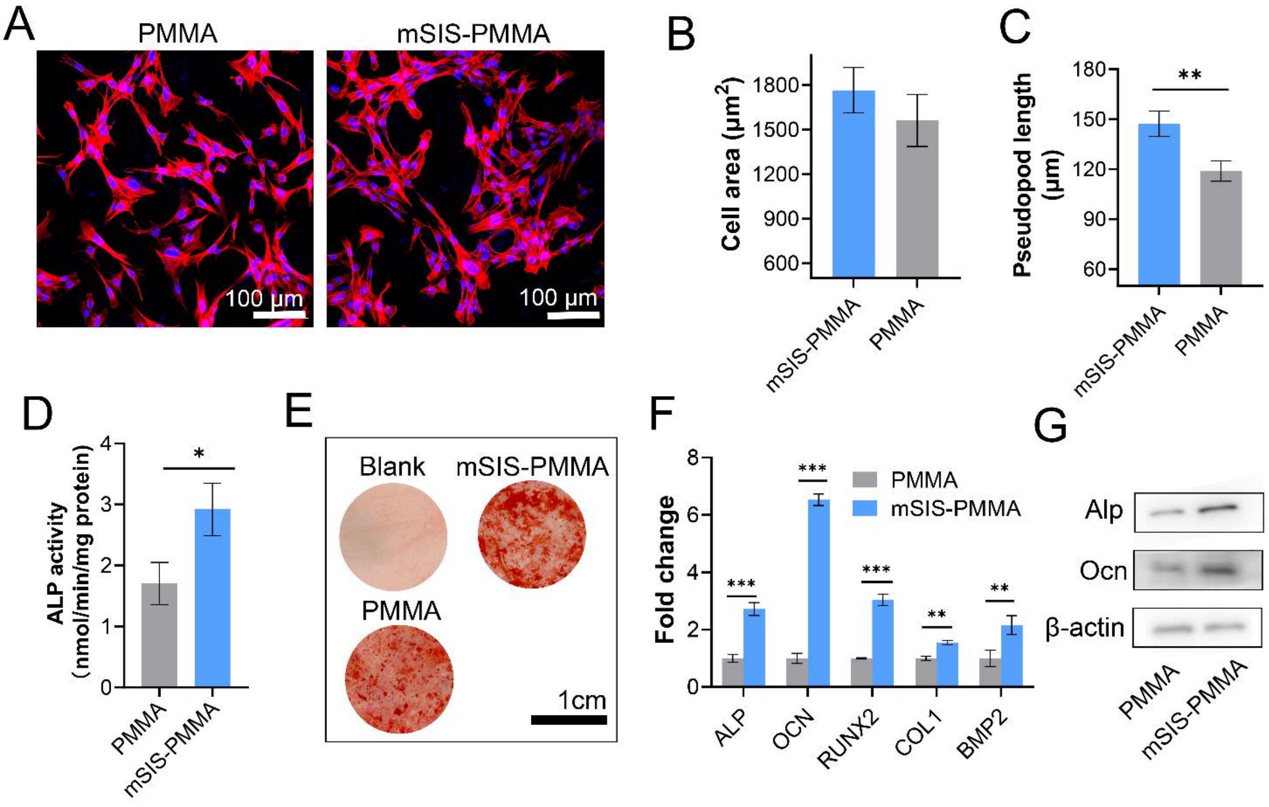

Figure 4. *In vitro* osteogenic activity of BMSCs. (A) BMSCs stained with rhodamine-phalloidin. (B) Comparison of cell area and (C) pseudopod length. (D) ALP activity in BMSCs on the cements. (E) Alizarin red staining. (F) Gene and (G) protein expression in BMSCs after 21 days of differentiation on the cements. Stats: mean ± SD, n = 5; *$P < 0.05$, **$P < 0.01$, ***$P < 0.001$.

3.4. Effect of mSIS–PMMA cement on bone regrowth in animals.

Mean bone mineral density in rabbits decreased by 23.03% after 8 weeks of dexamethasone sodium phosphate injection. Bone cements were injected into the L3 vertebrae of rabbits, and intraoperative observations were made (Fig. 5A). 3D-CT images showed mSIS–PMMA bone cement with irregular microporous structures due to mSIS degradation. In contrast, the exterior of the PMMA bone cement appeared smooth and intact (Figure 5B), implying that the utilization of mSIS–PMMA enhances the area of interaction with the adjacent bone tissue upon partial degradation *in vivo*. Based on the morphometric evaluation,

the mSIS–PMMA group exhibited a elevated percentage of peripheral bone volume (BV/TV = 19.14%, *P* = 0.001, *n* = 5) and greater peripheral trabeculae thickness (Tb.N = 0.73 mm^{-1}, *P* = 0.001, *n* = 5) compared with those of the control (BV/TV = 13.07%, Tb.N = 0.68 mm^{-1}, *n* = 5).

The bone healing efficacy was evaluated by staining sections of the treated vertebrae using methylene blue and magenta (Figure 5C). A negligible number of inflammatory cells was observed for both groups. The mSIS–PMMA group showed a significant enhancement in bone repair performance relative to the control group. Observation of the mSIS–PMMA group revealed new bone growth, characterized by an incomplete boundary with previously segmented bone tissues beginning to fuse and the emergence of a nascent bone structure. Nevertheless, the PMMA bone cement interface displayed a fine layer of fibrous tissue on its surface, and almost no new bone growth was observed. By employing Masson trichrome staining (Figure 5D), it was observed that the mSIS–PMMA bone cement exhibited an uneven demarcation, characterized by a green hue resulting from the infiltration of a substantial quantity of cartilage collagen. Collagen within the bone and calcified cartilage were stained with distinct colors. In contrast, the control exhibited a continuous, regular boundary with no new bone inside. The study demonstrated that the deterioration of mSIS–PMMA within the physiological milieu facilitated the infiltration of fresh host bone, thereby augmenting osseointegration. Moreover, histological assessment showed that the mSIS-PMMA group obtained substantially elevated scores in comparison to the PMMA group (Figure 5E–H).

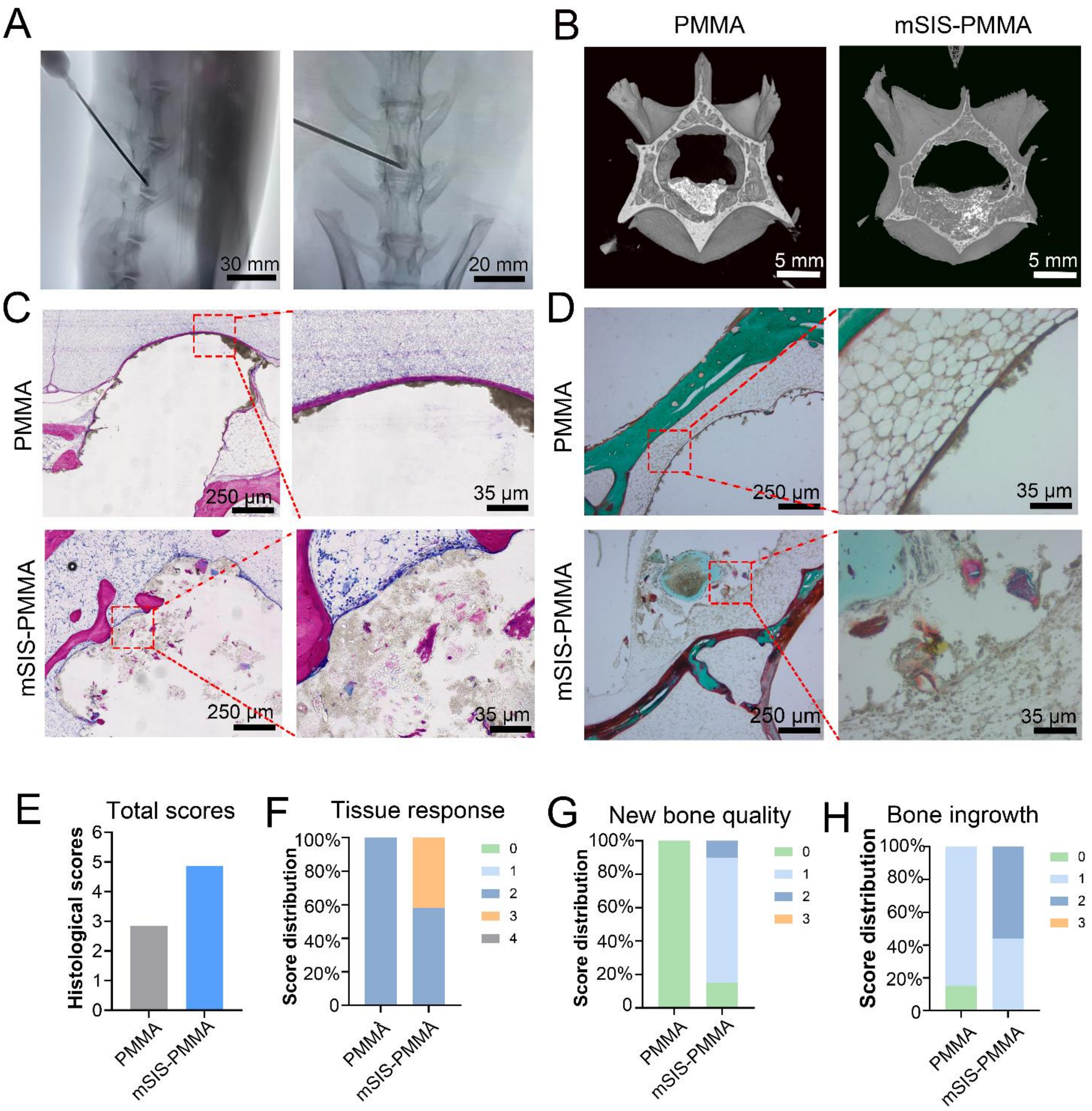

351 **Figure 5.** Histological analysis of bone ingrowth and regeneration in the rabbit osteoporosis model. (A) Intraoperative injection of the bone cement visualized by C-arm fluoroscopy. (B) Micro-CT images of vertebral bodies containing bone cement 6 weeks after bone cement injection. Histological staining images of hard tissue sections of the vertebral bodies of the PMMA and mSIS–PMMA groups 6 weeks after bone cement injection using (C) methylene blue and magenta and (D) Masson's trichrome staining. (E) The cumulative histological scores, encompassing (F) tissue response, (G) quality of new bone formation, and (H) degree of bone infiltration, were determined.

4. Discussion

PMMA bone cement is widely used for minimally invasive OVCF surgery, with over 40 brands on the market. However, all brands of PMMA bone cement used in OVCF treatment have a high elastic modulus, lack bioactivity, and are nondegradable *in vivo*. Incorporating active substances into PMMA bone cement can mitigate these shortcomings to a certain extent. As the active material degrades, it generates a porous structure and osteogenic microenvironment that enhance cell attachment and proliferation. This, in turn, augments the bonding surface between bone and bone cement while concurrently decreasing the elastic modulus [27]. As the main component of the active material mSIS, SIS has suitable biocompatibility, degradability, and mechanical properties for a bone cement additive and contains various growth factors. It can facilitate the growth of new bone tissue while reducing the modulus of elasticity. Moreover, nHA, a constituent of mSIS, exhibits osteoinductive properties and enhances the interaction between osteoblasts and the nearby microenvironment. This eventually leads to the promotion of fresh bone formation [28]. On this basis, mSIS was incorporated into the polymer powder of PMMA bone cement to produce a modified bone cement.

One important factor influencing clinical procedures is the required time for bone cement to harden. Curing times that are too long or too short are undesirable in clinical practice; excessively long curing times increase the risk of bone cement leakage, while excessively short ones reduce the handleability of bone cement. Although mSIS is insoluble in liquid MMA, it absorbs some liquid MMA during mixing, shortening curing time. However, the inclusion of a specific proportion of mSIS has an insignificant impact on the handling characteristics of the cement.

The higher elastic modulus of PMMA bone cement relative to cancellous bone results in stress shielding, which modifies stress transfer [29]. This can lead to bone tissue resorption

[30], increased osteoclast activity, and osteoblast apoptosis [31], which may increase the likelihood of fractures occurring in the surrounding vertebral body. Studies have shown that bone cements with low elastic moduli can effectively reduce stress levels in the neighboring bone [32]. Adding porogens to bone cement is a common method for reducing the elastic modulus. Aqueous sodium hyaluronate and hydroxypropylmethylcellulose have been added to PMMA powder to produce porous bone cement. This technique can significantly decrease the elastic modulus of bone cement. For example, by incorporating 50 vol.% hydroxyapatite, the elastic modulus of Vertecem® bone cement was diminished from 1,840 to 50 MPa, while the addition of 50 vol.% hydroxypropylmethylcellulose decreased the elastic modulus of Beracryl® bone cement from 2,800 to 120 MPa [33, 34]. In addition, Stryker® PMMA bone cement was modified with castor oil (5, 10, or 15 vol.%) [35]. Increasing the volume fraction of castor oil resulted in a lower modulus of elasticity and lower polymerization temperature but a longer curing time.

In the treatment of OVCF, bone cement plays crucial roles, such as restoring vertebral height, stabilizing the vertebral body, and providing mechanical support to the fractured vertebra. An ideal bone cement must have sufficient compressive strength and an appropriate elastic modulus to provide adequate support and reduce stress shielding [36]. Although incorporating active materials may decrease the elastic modulus of PMMA bone cement, it could potentially result in inadequate compressive strength, which is a concern. If the compressive strength is too low, the bone cement cannot support the fractured vertebral body when stress is applied at the cement–bone interface. For instance, the addition of isotonic saline to PMMA bone cement (Vertecem V+ Cement Kit; DePuy Synthes) induced a significant reduction in its modulus of elasticity, which decreased from 3,409 ± 312 to 1,131 ± 127 MPa. This was primarily attributed to an increase in microporosity. Consequently, the maximum compressive strength of the cement decreased from 86 ± 4 to 33 ± 3 MPa [37]. Lewin et al. [38] replaced some of the MMA monomers with linoleic acid and observed that

the elastic modulus of V-Steady® PMMA bone cement decreased from 3,360 ± 277 to 462 ± 78 MPa after 24 h of curing, while the compressive strength decreased to 12 ± 1 MPa. Although these studies have made progress in reducing the modulus of elasticity of bone cements, the inherently low compressive strength of the resultant bone cements makes them unsuitable for orthopedic surgery. Compared with the bone cements from these studies, mSIS–PMMA exhibited a reduced modulus of elasticity with reasonably high compressive strength. In this study, the inclusion of mSIS into PMMA bone cement decreased the elastic modulus from 2,572 ± 4.8 to 263.7 ± 3.7 MPa at a ratio of 15:85. This material exhibits an elastic modulus range that is similar to the trabecular architecture present in human bones. In addition, the mSIS–PMMA (mass ratio of mSIS:PMMA = 15:85) bone cement still had a compressive strength of more than 70 MPa, sufficient to provide support to the affected vertebral body. The low elastic modulus of mSIS–PMMA relative to that of conventional PMMA bone cement, combined with adequate mechanical strength and handleability, can reduce postoperative complications resulting from stress shielding between the bone cement and cancellous bone.

As a biologically inert material, PMMA bone cement does not promote cell proliferation and adhesion. To improve the bioactivity of bone cement, active materials have been introduced to PMMA. For instance, incorporating mineralized collagen into Mendec Spine® PMMA bone cement enhanced its biocompatibility, and the resulting modified bone cement stimulated the attachment, growth, and bone-forming ability of osteoblasts [26]. Another study modified the same PMMA bone cement by replacing a portion of the bone cement powder with tricalcium silicate. *In vitro* cell culture tests showed that the bone cement's pro-proliferative effect tended to increase with higher tricalcium silicate content [39]. Biodegradable bone cement was obtained by incorporating Mg microspheres into Alfa Aesar® PMMA bone cement. *In vitro* cellular experiments showed improved cell proliferation when exposed to the modified bone cement surface [40]. Although the above studies have achieved

higher biological activity, their modification of biological activity and mechanical properties is unbalanced. In contrast, the mSIS–PMMA reported in the present study exhibits simultaneous improvements in biological activity and mechanical properties, representing a more promising bone cement for clinical applications.

The initial stage of the biological response to implanted biomaterials *in vivo* is cell attachment, which is greatly influenced by the physicochemical properties of the implant surface [41]. Our *in vitro* cellular experiments demonstrated improved cell growth with extended morphology and intercellular connections forming a mesh-like structure following exposure to mSIS–PMMA bone cement. A hydrophilic surface is more conducive to cell attachment and growth compared with a hydrophobic surface [42], implying that the hydrophilic surface of mSIS–PMMA bone cement may enhance cell attachment. Moreover, cells cultured on mSIS–PMMA bone cement exhibited stronger osteogenic differentiation, as indicated by increased osteogenic gene expression. These findings suggest that mSIS may enhance osteogenic activity, which is critical for new bone formation. This may be because of the ability of SIS to activate the BMP signaling pathway [22] and the ability of nHA, the main degradation product of mSIS in the physiological environment, to bind to proteins such as ALP and OCN in osteoblasts, thus promoting osteogenic cell differentiation [43]. The *in vitro* cellular experiments we conducted suggest that mSIS–PMMA bone cement possesses both osteoconductive and osteoinductive properties, which could potentially promote the integration of the bone cement into the affected spinal segment.

Conventional PMMA bone cement does not integrate with the bone tissue after implantation into a fractured vertebral body [44]. This is because PMMA cannot be degraded into small molecules *in vivo*, and the dense internal structure does not provide space for new bone ingrowth [45]. Instead, PMMA bone cement may be encapsulated by fibrous tissue. Furthermore, the low attachment strength between the bone cement and surrounding bone [46] can cause slight movement of the cement [47], resulting in the formation of small

particles or even dislodgment of the cement [48]. In contrast, bioactive bone cement has been shown to have excellent osseointegrative properties [49]. Biologically active bone cements can react with human body fluids in the early stage of transplantation to generate nHA at the bone–tissue interface and form organic bonds. Subsequently, nHA induces osteoblast matrix mineralization to generate new bone. Synostosis between the new bone and bone cement improves interfacial integration [50]. The experimental results demonstrate that the mSIS in mSIS–PMMA bone cement gradually degrades in the physiological environment, forming a microporous structure capable of supporting cell ingrowth. Moreover, the excellent biocompatibility of mSIS–PMMA bone cement and the local ionic microenvironment with high P and Ca levels due to the degradation of mSIS can promote cell attachment and proliferation, recruit osteoblasts, and facilitate cell migration. These effects jointly promote the integration of bone tissue and bone cement within the vertebral bone, making it safer for clinical use.

Overall, the partially biodegradable mSIS–PMMA bone cement prepared here has good handleability and provides excellent support, while its elastic modulus closely resembles that of human cancellous bone in contrast to that of conventional bone cement. Additionally, the biocompatible, bioactive, and biodegradable characteristics of mSIS-PMMA bone cement can promote the formation of fresh bone tissue, while simultaneously providing structural support to the affected vertebra. Incorporating mSIS into PMMA bone cement holds promising clinical implications for percutaneous kyphoplasty and percutaneous vertebroplasty procedures.

Acknowledgments

The authors thank the Youth Innovation Project of the Project of Pharmaceutical Health Science and Technology Program of Zhejiang Province [2023RC027], the Natural Science Foundation of Ningbo [202003N4281], and the Youth Talent Cultivation Program of the

Affiliated Hospital of Medical School of Ningbo University [FYQM-LC-202005] for financial support.

References

[1] Ji C, Rong Y, Wang J, Yu S, Yin G, Fan G, et al. Risk factors for refracture following primary osteoporotic vertebral compression fractures. *Pain Phys* **2021**;24:E335–E340, DOI: 10.1016/j.bas.2021.100253.

[2] Chen X, Hu Y, Geng Z, Su J. The “three in one” bone repair strategy for osteoporotic fractures. *Front Endocrinol* (Lausanne) **2022**;13:910602, DOI: 10.3389/fendo.2022.910602.

[3] Zhao R, Shang T, Yuan B, Zhu X, Zhang X, Yang X. Osteoporotic bone recovery by a bamboo-structured bioceramic with controlled release of hydroxyapatite nanoparticles. *Bioact Mater* **2022**;17:379–393, DOI: 10.1016/j.bioactmat.2022.01.007.

[4] Inose H, Kato T, Ichimura S, Nakamura H, Hoshino M, Takahashi S, et al. Factors contributing to residual low back pain after osteoporotic vertebral fractures. *J Clin Med* **2022**;11:1566, DOI: 10.3390/jcm11061566.

[5] Weber A, Huysmans SMD, van Kuijk SMJ, Evers SMAA, Jutten EMC, Senden R, et al. Effectiveness and cost-effectiveness of dynamic bracing versus standard care alone in patients suffering from osteoporotic vertebral compression fractures: protocol for a multicentre, two-armed, parallel-group randomised controlled trial with 12 months of follow-up. *BMJ* (Open) **2022**;12:e054315, DOI: 10.1136/bmjopen-2021-054315.

[6] Cheung WH, Wong RMY, Choy VMH, Li MCM, Cheng KYK, Chow SKH. Enhancement of osteoporotic fracture healing by vibration treatment: the role of osteocytes. *Injury* **2021**;52:S97–S100, DOI: 10.1016/j.injury.2020.05.020.

[7] Vellucci R, Terenzi R, Kanis JA, Kress HG, Mediati RD, Reginster JY, et al. Understanding osteoporotic pain and its pharmacological treatment. *Osteoporos Int* **2018**;29:1477–1491, DOI: 10.1007/s00198-018-4476-y.

[8] Park JS, Park YS. Survival analysis and risk factors of new vertebral fracture after vertebroplasty for osteoporotic vertebral compression fracture. *Spine J* **2021**;21:1355–1361, DOI: 10.1016/j.spinee.2021.04.022.

[9] Patel N, Jacobs D, John J, Fayed M, Nerusu L, Tandron M, et al. Balloon kyphoplasty vs vertebroplasty: A systematic review of height restoration in osteoporotic vertebral compression fractures. *J Pain Res* **2022**;15:1233–1245, DOI: 10.2147/JPR.S344191.

[10] Dai C, Liang G, Zhang Y, Dong Y, Zhou X. Risk factors of vertebral re-fracture after PVP or PKP for osteoporotic vertebral compression fractures, especially in Eastern Asia: a systematic review and meta-analysis. *J Orthop Surg Res* **2022**;17:161, DOI: 10.1186/s13018-022-03038-z.

[11] Rabei R, Patel K, Ginsburg M, Patel MV, Turba UC, Arslan B, et al. Percutaneous vertebral augmentation for vertebral compression fractures: national trends in the medicare population (2005–2015). *Spine* **2019**;44:123–133, DOI: 10.1097/BRS.0000000000002893.

[12] Miao F, Zeng X, Wang W, Zhao Z. Percutaneous vertebroplasty with high- versus low-viscosity bone cement for osteoporotic vertebral compression fractures. *J Orthop Surg Res* **2020**;15:302, DOI: 10.1186/s13018-020-01835-y.

[13] Xiong YC, Guo W, Xu F, Zhang CC, Liang ZP, Wu L, et al. Refracture of the cemented vertebrae after percutaneous vertebroplasty: risk factors and imaging findings. *BMC Musculoskelet Disord* **2021**;22:459, DOI: 10.1186/s12891-021-04355-w.

[14] Liu X, Cheng C, Peng X, Xiao H, Guo C, Wang X, et al. A promising material for bone repair: PMMA bone cement modified by dopamine-coated strontium-doped

calcium polyphosphate particles. *R Soc Open Sci* **2019**;6:191028, DOI: 10.1098/rsos.191028.

[15] Zhu J, Zhang K, Luo K, Qiu Z, Yang S, Cui F, et al. Mineralized collagen modified polymethyl methacrylate bone cement for osteoporotic compression vertebral fracture at 1-year follow-up. *Spine* **2019**;44:827–838, DOI: 10.1097/BRS.0000000000002971.

[16] Phakatkar AH, Shirdar MR, Qi ML, Taheri MM, Narayanan S, Foroozan T, et al. Novel PMMA bone cement nanocomposites containing magnesium phosphate nanosheets and hydroxyapatite nanofibers. *Mater Sci Eng C Mater Biol Appl* **2020**;109:110497, DOI: 10.1016/j.msec.2019.110497.

[17] Yang L, Zhou J, Yu K, Yang S, Sun T, Ji Y, et al. Corrigendum to "Surface modified small intestinal submucosa membrane manipulates sequential immunomodulation coupled with enhanced angio- and osteogenesis towards ameliorative guided bone regeneration" [Mater. Sci. Eng. C. 119 (2021) 111641]. *Biomater Adv* **2022**;139:212970, DOI: 10.1016/j.msec.2020.111641.

[18] Zhao P, Li X, Fang Q, Wang F, Ao Q, Wang X, et al. Surface modification of small intestine submucosa in tissue engineering. *Regen Biomater* **2020**;7:339–348, DOI: 10.1093/rb/rbaa014

[19] Su Y, Ye B, Zeng L, Xiong Z, Sun T, Chen K, et al. Small intestinal submucosa biomimetic periosteum promotes bone regeneration. *Membranes* (Basel) **2022**;12:719, DOI: 10.3390/membranes12070719.

[20] Zhang L, Ma S, Wei P, Zhao Y, Mu Y, Wu J, et al. Small intestinal submucosa membrane modified by fusion peptide-mediated extracellular vesicles to promote tissue regeneration. *Adv Healthc Mater* **2021**;10:e2101298, DOI: 10.1002/adhm.202101298.

[21] Zhang C, Zhu J, Zhang T, Li M, Jiang G, Zhao J. Small intestinal submucosa/polymethyl methacrylate composite bone cement for vertebral repair. *Mater Des* **2018**;154:254–265, DOI: 10.1016/j.matdes.2018.05.045.

[22] Li M, Zhang C, Cheng M, Gu Q, Zhao J. Small intestinal submucosa: A potential osteoconductive and osteoinductive biomaterial for bone tissue engineering. *Mater Sci Eng C Mater Biol Appl* **2017**;75:149–156, DOI: 10.1016/j.msec.2017.02.042

[23] Yang M, Wang J, Zhu Y, Mao C. Bio-templated growth of bone minerals from modified simulated body fluid on nanofibrous decellularized natural tissues. *J Biomed Nanotechnol* **2016**;12:753–761, DOI: 10.1166/jbn.2016.2202.

[24] Yao S, Jin B, Liu Z, Shao C, Zhao R, Wang X, et al. Biomineralization: From material tactics to biological strategy. *Adv Mater* **2017**;29:14, DOI: 10.1002/adma.201605903.

[25] Riau AK, Lwin NC, Gelfand L, Hu H, Liedberg B, Chodosh J, et al. Surface modification of corneal prosthesis with nano-hydroxyapatite to enhance in vivo biointegration. *Acta Biomater* **2020**;107:299–312, DOI: 10.1016/j.actbio.2020.01.023.

[26] J. Zhu, S. Yang, K. Cai, S. Wang, Z. Qiu, J. Huang et al. Bioactive poly (methyl methacrylate) bone cement for the treatment of osteoporotic vertebral compression fractures. *Theranostics* **2020**, 10, 6544–6560, DOI: 10.7150/thno.44428.

[27] Tomazela L, Cruz MAE, Nascimento LA, Fagundes CC, da Veiga MAMS, Zamarioli A, et al. Fabrication and characterization of a bioactive polymethylmethacrylate-based porous cement loaded with strontium/calcium apatite nanoparticles. *J Biomed Mater Res A* **2022**;110:812–826, DOI: 10.1002/jbm.a.37330.

[28] Wang J, Wang M, Chen F, Wei Y, Chen X, Zhou Y, et al. Nano-hydroxyapatite coating promotes porous calcium phosphate ceramic-induced osteogenesis via BMP/Smad signaling pathway. *Int J Nanomedicine* **2019**;14:7987–8000, DOI: 10.2147/IJN.S216182.

[29] Leschinger T, Engel K, Brüggemann GP, Dederer V, Neiss WF, Scheyerer MJ, et al. Glass-polyalkenoate cement: an alternative material for kyphoplasty in osteoporotic vertebral compression fractures – an ex vivo study. *J Mech Behav Biomed Mater* **2018**;83:46–51, DOI: 10.1016/j.jmbbm.2018.03.008.

[30] Gao X, Ge J, Zhou W, Xu L, Geng D. IL-10 inhibits osteoclast differentiation and osteolysis through MEG3/IRF8 pathway. *Cell Signal* **2022**;95:110353, DOI: 10.1016/j.cellsig.2022.110353.

[31] Bu YM, Zheng DZ, Wang L, Liu J. Abrasive endoprosthetic wear particles inhibit IFN-γ secretion in human monocytes via upregulating TNF-α-induced miR-29b. Inflammation **2017**;40:166–173, DOI: 10.1007/s10753-016-0465-5.

[32] Zhu J, Yang S, Yang Y, Yao T, Liu G, Fan S, et al. Modified poly(methyl methacrylate) bone cement in the treatment of Kümmell disease. *Regen Biomater* **2021;**8:1-9, DOI: 10.1093/rb/rbaa051

[33] Boger A, Bisig A, Bohner M, Heini P, Schneider E. Variation of the mechanical properties of PMMA to suit osteoporotic cancellous bone. *J Biomater Sci Polym Ed* **2008**;19:1125–1142, DOI: 10.1163/156856208785540154.

[34] Boger A, Bisig A, Bohner M, Heini P, Verrier S, Schneider E. Properties of an injectable low modulus PMMA bone cement for osteoporotic bone. *J Biomed Mater Res B Appl Biomater* **2008**;86:474–482, DOI: 10.1002/jbm.b.31044.

[35] Tai CL, Lai PL, Lin WD, Tsai TT, Lee YC, Liu MY, et al. Modification of mechanical properties, polymerization temperature, and handling time of polymethylmethacrylate cement for enhancing applicability in vertebroplasty. *BioMed Res Int* **2016**;2016:7901562, DOI: 10.1155/2016/7901562.

[36] Aghyarian S, Bentley E, Hoang TN, Gindri IM, Kosmopoulos V, Kim HKW, et al. In vitro and in vivo characterization of premixed PMMA-CaP composite bone cements. *ACS Biomater Sci Eng* **2017;**3:2267–2277, DOI: 10.1021/acsbiomaterials.7b00276.

[37] Schröder C, Nguyen M, Kraxenberger M, Chevalier Y, Melcher C, Wegener B, et al. Modification of PMMA vertebroplasty cement for reduced stiffness by addition of normal saline: a material properties evaluation. *Eur Spine J* **2017**;26;3209–3215, DOI: 10.1007/s00586-016-4845-1

[38] Lewin S, Försth P, Persson C. Low-modulus PMMA has the potential to reduce stresses on endplates after cement discoplasty. *J Funct Biomater* **2022**;13:18, DOI: 10.3390/jfb13010018.

[39] Wei Y, Baskaran N, Wang HY, Su YC, Nabilla SC, Chung RJ. Study of polymethylmethacrylate/tricalcium silicate composite cement for orthopedic application. *Biomed J* **2022**;S2319–4170:00090–00097, DOI: 10.1016/j.bj.2022.05.005.

[40] Zhai Q, Han F, He Z, Shi C, Zhou P, Zhu C, et al. The "magnesium sacrifice" strategy enables PMMA bone cement partial biodegradability and osseointegration potential. *Int J Mol Sci* **2018**;19:1746, DOI: 10.3390/ijms19061746.

[41] Majhy B, Priyadarshini P, Sen AK. Effect of surface energy and roughness on cell adhesion and growth – facile surface modification for enhanced cell culture. *RSC Adv* **2021**;11:15467–15476, DOI: 10.1039/d1ra02402g.

[42] Paterlini TT, Nogueira LFB, Tovani CB, Cruz MAE, Derradi R, Ramos AP. The role played by modified bioinspired surfaces in interfacial properties of biomaterials. *Biophys Rev* **2017**;9:683–698, DOI: 10.1007/s12551-017-0306-2.

[43] Wang M, Sa Y, Li P, Guo Y, Du Y, Deng H, et al. A versatile and injectable poly(methyl methacrylate) cement functionalized with quaternized chitosan-glycerophosphate/nanosized hydroxyapatite hydrogels. *Mater Sci Eng C Mater Biol Appl* **2018**;90:264–272, DOI: 10.1016/j.msec.2018.04.075.

[44] Aghyarian S, Hu X, Haddas R, Lieberman IH, Kosmopoulos V, Kim HKW, et al. Biomechanical behavior of novel composite PMMA-CaP bone cements in an

anatomically accurate cadaveric vertebroplasty model. *J Orthop Res* **2017**;35:2067–2074, DOI: 10.1002/jor.23491.

[45] He Q, Chen H, Huang L, Dong J, Guo D, Mao M, et al. Porous surface modified bioactive bone cement for enhanced bone bonding. *PLOS ONE* **2012**;7:e42525, DOI: 10.1371/journal.pone.0042525.

[46] Kong J, Ma J, Wu Z, Wang H, Peng X, Wang S, et al. Minimally invasive injectable lumbar interbody fusion with mineralized collagen-modified PMMA bone cement: A new animal model. *J Appl Biomater Funct Mater* **2020**;18:2280800020903630, DOI: 10.1177/2280800020903630.

[47] Liu H, Liu B, Gao C, Meng B, Yang H, Yu H, et al. Injectable, biomechanically robust, biodegradable and osseointegrative bone cement for percutaneous kyphoplasty and vertebroplasty. *Int Orthop* **2018**;42:125–132, DOI: 10.1007/s00264-017-3674-0.

[48] Wang Y, Shen S, Hu T, Williams GR, Bian Y, Feng B, et al. Layered double hydroxide modified bone cement promoting osseointegration via multiple osteogenic signal pathways. *ACS Nano* **2021**;15:9732–9745, DOI: 10.1021/acsnano.1c00461.

[49] Sun X, Wu Z, He D, Shen K, Liu X, Li H, et al. Bioactive injectable polymethylmethacrylate/silicate bioceramic hybrid cements for percutaneous vertebroplasty and kyphoplasty. *J Mech Behav Biomed Mater* **2019**;96:125–135, DOI: 10.1016/j.jmbbm.2019.04.044.

[50] Hench LL, Thompson I. Twenty-first century challenges for biomaterials. *J R Soc Interface* **2010**;7Suppl 4;S379–S391, DOI: 10.1098/rsif.2010.0151.focus.

7% Overall Similarity

Top sources found in the following databases:

- 6% Internet database
- 6% Publications database
- Crossref database
- Crossref Posted Content database
- 0% Submitted Works database

TOP SOURCES

The sources with the highest number of matches within the submission. Overlapping sources will not be displayed.

#	Source	Similarity
1	thno.org<br>Internet	1%
2	science.gov<br>Internet	<1%
3	Yi Chai, He Zhao, Shuhui Yang, Xiaohan Gao et al. "Structural alignmen...<br>Crossref	<1%
4	Li, Tao, Xisheng Weng, Yanyan Bian, Lei Zhou, Fuzhai Cui, and Zhiye Qi...<br>Crossref	<1%
5	academic.oup.com<br>Internet	<1%
6	mdpi.com<br>Internet	<1%
7	Wang, Jian-Hua. "Infective severe acute pancreatitis: A comparison of ...<br>Crossref	<1%
8	Tianli Wu, Hui Tang, Jianghua Yang, Zhihao Yao, Long Bai, Yuping Xie, ...<br>Crossref	<1%

9	nature.com<br>Internet	<1%
10	frontiersin.org<br>Internet	<1%
11	jeccr.biomedcentral.com<br>Internet	<1%
12	purehost.bath.ac.uk<br>Internet	<1%
13	Shuhui Yang, Jinjin Zhu, Changfeng Lu, Yi Chai et al. "Aligned fibrin/fun...<br>Crossref	<1%
14	Zhiwei Jie, Ziang Xie, Wenbin Xu, Xiangde Zhao et al. "SREBP-2 aggrav...<br>Crossref	<1%
15	Chi Zhang, Jinjin Zhu, Tingxia Zhang, Mei Li, Guoqiang Jiang, Jiyuan Zh...<br>Crossref	<1%
16	Elena Rusu, Marioara Onciu. "Polycondensates of 2'-(Chalcone-4-Oxy)E...<br>Crossref	<1%
17	Yuan-Ning Zhang, Ruibo Zhao, Jie Cao, Bowen Chen, Dandan Luo, Jiaju...<br>Crossref	<1%
18	Cairong Li, Jianqiao Sun, Keda Shi, Jing Long, Long Li, Yuxiao Lai, Ling ...<br>Crossref	<1%
19	Duoyi Zhao, Tongtong Zhu, Jie Li, Liguo Cui, Zhiyu Zhang, Xiuli Zhuang,...<br>Crossref	<1%
20	deepblue.lib.umich.edu<br>Internet	<1%

21	iopscience.iop.org<br>Internet	<1%
22	Handbook of Bioceramics and Biocomposites, 2016.<br>Crossref	<1%
23	Lele Bao, Xiangjun Li, Yingmeng Qi, Zhenwei Wang, Jun Li. "PEG/SBA-1...<br>Crossref	<1%
24	Shiqing Ma, Yilin Yang, Yuzhu Mu, Huizhen Peng et al. "Modification of ...<br>Crossref	<1%
25	Zhao Yang, Lei Chen, Yuxin Hao, Yuan Zang et al. "Synthesis and Chara...<br>Crossref	<1%
26	docksci.com<br>Internet	<1%
27	hdl.handle.net<br>Internet	<1%
28	juniperpublishers.com<br>Internet	<1%
29	mdpi-res.com<br>Internet	<1%
30	researchgate.net<br>Internet	<1%
