# Supplementary material for: Bioactive mineralized small intestinal submucosa acellular matrix/PMMA bone cement for vertebral bone regeneration
Source: Regen Biomater. 2023 May 11;10:rbad040. doi: 10.1093/rb/rbad040 (PMC10224805; doi:10.1093/rb/rbad040)
Supplement: rbad040_Supplementary_Data [file rbad040_supplementary_data.zip › _____~1.PDF]

通知书

申请人 蒋国强

你提交的项目： 矿化小肠粘膜下层复合PMMA骨水泥的制备及其分子机制研究 （申请编号： 11473 ）中关于动物实验的方案，经我委员会审核，符合要求，予以初审通过。 动物实验时间为： 2022-09-11 ~ 2022-12-15

特此通知。

（注：此通知书仅限于实验方案使用，不得用作其他地方。）

宁波大学实验动物伦理委员会

2022 年 10 月 09 日
