# Supplementary material for: Bioactive mineralized small intestinal submucosa acellular matrix/PMMA bone cement for vertebral bone regeneration
Source: Regen Biomater. 2023 May 11;10:rbad040. doi: 10.1093/rb/rbad040 (PMC10224805; doi:10.1093/rb/rbad040)
Supplement: rbad040_Supplementary_Data [file rbad040_supplementary_data.zip › Respond to Decision Letter.doc]

Dear Editors and Reviewers:

We really appreciate the opportunity given by the editor to revise our manuscript entitled “Bioactive mineralized small intestinal submucosa acellular matrix/PMMA bone cement for vertebral bone regeneration” (ID: RB-2022-269). The comments from the reviewers are constructive and valuable to improve the quality of our paper. We would like to confirm ALL the comments have been considered and addressed to the best of our knowledge. Below, please find the comments in black and our responses in red. The changes in the manuscript are also presented in yellow font.

Responses to reviewers:

**Specific Comments:**

The reviewer(s) have recommended publication, but also suggest some major revisions to your manuscript. Specific points that the authors need to address are as follows:

**Reviewer #1:**

**Comment 1:** In Table 1, the contrast agent is "BaSO4/ZrO2", but as I know, the commercialized Mendec Spine PMMA bone cements only use BaSO4 as the contrast agent. Please check the composition of the product.

**Response 1:** We sincerely thank the reviewer for his/her careful reading. As suggested by the reviewer, we have checked the composition of the product and corrected “BaSO4/ZrO2” to “Barium sulphate ” in Table 1.

**Comment 2:** Unit must be provided for the x-coordinate in Figure 1B. Although I know the unit is "minutes". This unit is not introduced in the context.

**Response 2:** We appreciate the reviewer’s attention to detail; we have added the unit for the x axis in Figure 1C.

**Comment 3:** In the FTIR spectra shown by Figure C. Which group of the mSIS-PMMA was used for the characterization? The ration of mSIS to PMMA was not provided in related Materials and Methods section. **Response 3:** We apologize for the confusion. The ratio of mSIS to PMMA is not specified here because the characteristic peaks of mSIS appear in the same position for mSIS-PMMA with different mSIS ratios. According to the reviewer's suggestion, we have added the ratio of mSIS to PMMA in line 119 on page 5.

**Comment 4:** As a key component for the modification of the PMMA bone cement, more information of the mSIS should be provided. "2.1 Materials synthesis" is too simple to provide enough information on mSIS. What was the morphology/shape of the mSIS used for the modification? Particles? Powders? Or other forms. In similar studies, the component for the modification were usually powders or small particles. So, if mSIS was particles/powders, what was the size distribution?

**Response 4:** We thank the reviewer for this insightful comment. We have added relevant information about the synthesis, properties, and size distribution of mSIS to section “2.1 Materials synthesis”.

**Comment 5:** The authors mentioned other modification studies on PMMA bone cements by other biomaterials, such as mineralized collagen, tricalcium silicate, etc. Could the authors compare current study to those similar studies?

**Response 5:** Thank you for your insightful question. We compared our results with those of previous studies, such as mineralized collagen and tricalcium silicate, in the discussion section and highlighted the changes in yellow.

**Reviewer #2:**

**Comment 1:** The degradation performance of the mSIS-PMMA cement is important for its mechanical and biological properties, so the relevant characterizations should be performed.

**Response 1:** Thank you for pointing this out. According to the reviewer's suggestion, we have carried out additional experiments to measure the degradation of mSIS–PMMA and added a description of the procedure and results to the revised manuscript.

**Comment 2:** Are any other blank group, without co-culturing with the cement, used as a control in vitro studies? This should be discussed with the material groups, if any.

**Response 2:** We thank the reviewer for this insightful comment. A blank petri dish of BMSCs without co-culturing with any cement was used as a blank and its results after Calcein AM/PI staining, CCK-8 test and immunofluorescence staining are shown in Figure A, B and C, respectively in the below figure. Compared to the PMMA and mSIS-PMMA groups, the blank group showed the most live cells (green in Figure A), the greatest absorbance (Figure B) and the highest cell density with the most cellular pseudopods (Figure C).

In the manuscript, we decided not to show the blank group results and use PMMA as a control for the following reasons:

1. The purpose of setting a control group is to eliminate the influence of irrelevant variables and enhance the reliability of the experimental results. By using PMMA as a control, adding mSIS is a single variable when comparing mSIS-PMMA with PMMA. The influence of mSIS is evident.

2. The results of blank group suggests that PMMA has no biological activity, which has been confirmed by many studies, and relevant literature is cited in this paper.

3.Including blank group may also mislead readers. Petri dishes have excellent biological activity, and by using it as a blank to compare with mSIS-PMMA, readers may draw adverse conclusions about mSIS-PMMA.

 (A) BMSCs viability of blank group, PMMA and mSIS–PMMA bone cement using live (green)/dead (red) staining. (B) OD values of BMSCs cultured in blank group, PMMA and mSIS–PMMA bone cement extracts; *n* = 5; **P* < 0.05, ***P* < 0.01, and ****P* < 0.001. (C) Morphology of BMSCs on days 1, 3, 5, and 7 in blank group, PMMA and mSIS–PMMA bone cements. Cells stained with rhodamine-phalloidin for F-actin (red) and DAPI for nuclei (blue).
